# Forensic Medicine

**Published:** 1838-07

**Authors:** 


### FORENSIC MEDICINE.

### *Murder by Carbonic Acid.* By M. DEVERGIE.

[THE rare circumstance of murder being attempted or perpetrated by an aërial poison has induced us to select the following case, as one possessing some medico-legal interest.]

In February, 1836, a woman residing in the Rue Descartes, Paris, was found dead in her apartment under suspicious circumstances. Her husband was accused of having murdered her. The statement which he made relating to the death of his wife, was that they both wished to destroy themselves. After their usual evening meal they retired to their room, which was small and ill-ventilated. A charcoal-fire was lighted in the middle of the room, and they placed themselves close to it, so as to inhale the vapour of the burning fuel. The deceased fell asleep, and in the course of a few hours he found that she was quite dead: while he himself was not affected by the vapour. He procured more charcoal, and continued to burn it in the room, still closed up, for five days and five nights successively, sitting, during that time, by the side of the body of his wife. He took no food and only a small quantity of liquid to satisfy his thirst. He occasionally had two charcoal-fires lighted at once, for the purpose of producing more vapour; and placed himself between them, in order more effectually to suffocate himself, but without success. Such was the account given by the prisoner. The charge laid against him, was, that he had destroyed his wife and that he had concealed her body in the room for four or five days.

The medical men to whom the investigation was intrusted, found the body of the deceased on the floor of the room with the head reclining against the foot of the bed. It was much putrefied. The face was swollen and of a greenish black colour, an appearance which was also observed on the trunk and limbs. The skin of the neck was puffy and discoloured: there was no trace of any impression of a ligature, nor were there any marks of violence. On a close inspection, no violence or mechanical injury was to be seen either externally or internally. The viscera were in a high state of decomposition: the lungs were gorged with blood. The contents of the stomach, on analysis, presented no trace of poison. The quantity of food found in the stomach, however, was not such as to correspond with the prisoner's declaration, that his wife had made a full meal just before her death.

Several marks of violence but of an unimportant nature, were found on the person of the *prisoner.* The medico-legal questions required to be answered were as follows:

1. *Whether the room in which the deceased's body was found was such as to allow of her being suffocated by charcoal-vapour, in the manner stated by the husband?*

This question was answered in the affirmative, for the following reasons: Although the apartment was not so perfectly closed as entirely to exclude the external air; yet, as the witnesses very properly contended, this condition is by no means necessary to death from carbonic acid. If the access of air were even more free than it was shewn to have been in this case, still so soon as the air of a room becomes impregnated with the gas in the proportion of about ten per cent. by volume, that is enough to bring on all the effects of poisoning or suffocation. Thus, then, even a door or a window of an apartment might be partially open, and yet an individual perish from this cause. In the case of the deceased the closure of the room, judging from the prisoner's own description, was sufficiently perfect easily to account for her death in the manner supposed.

2. *Whether it was possible that the deceased should have died from this form of asphyxia, without the prisoner himself at the same time experiencing the deleterious effects of the gas?*

A decided answer in the negative was returned, for the reason that carbonic acid is equally fatal, when respired, to both sexes,—to man as well as to animals. It was admitted that two persons equally exposed might not experience the noxious effects in the same degree: but it was impossible to allow under the well-known properties of this gas, that of two equally exposed, one should perish from it in about three hours, while the other should not experience the least ill consequences. It was also highly improbable that a man, in the course of four or five days, should have consumed upwards of five bushels of charcoal (the quantity stated by the prisoner) without suffering any serious symptoms; and this in an apartment in which, according to his own declaration, a quarter of a bushel had sufficed to destroy his wife in a few hours. The improbability of the story was strengthened by the consideration that this large quantity of charcoal was not consumed in small portions: but that two fires were kept burning at once, so that the slight ventilation which might have taken place through the crevices in the room, could not have prevented a speedy and fatal contamination of the air. Either the man himself must therefore have suffered from the symptoms of asphyxia or his statement was false.

3. *Whether the advanced state of putrefaction within less than five days after death, confirmed or invalidated the supposition of death by asphyxia?*

This question seems to have been answered somewhat evasively. The witness admits what is generally believed by medical jurists, that, in death from asphyxia, putrefaction is commonly much retarded. This is ascribed by him to the antiseptic properties of carbonic acid when absorbed into the blood. On the whole, he thinks that the advanced state of putrefaction in the deceased, dying as she did in the month of February, is repugnant to the supposition of her having been asphyxiated by carbonic acid.

4. *Whether if death took place from asphyxia, the skin of the deceased should not have appeared livid or red in spite of the putrefactive process?*

In answer to this question, it is alleged by the witness (M. Devergie) that the red colour of the skin in asphyxiated subjects remains for a long time visible, notwithstanding the access of putrefaction. He therefore considers it surprising that it was not found on the skin of the deceased, if she had really been asphyxiated. In regard to the question whether this appearance be a constant accompaniment of asphyxia, he states that in eight years' experience he has never failed to meet with it in asphyxiated subjects.

5. *Do females resist the effects of such an asphyxiated medium longer than males?*

To illustrate this enquiry a few statistical facts are mentioned. During the years 1834-5, there were in Paris 360 cases of death from asphyxia by carbonic acid. Of this number there were nineteen cases of two persons together, male and female. There were only three instances out of these in which one of the two could be resuscitated; and in each instance it was the *female.* The witness was inclined to think, not only from this but from other observations, that females are better capable of resisting the asphyxiating effects of carbonic acid than males.

6. *Whether asphyxia, under such circumstances, takes place most readily in individuals who are placed in contact with the floor or soil?*

If, observes the witness, a stratum of carbonic acid had collected on the floor of the apartment, this could only have happened after the entire combustion of the whole of the charcoal, and when a perfectly uniform temperature had become established throughout the whole mass of air in the room. Asphyxia must have taken place long before these conditions could have existed. The prisoner and the deceased were not therefore, by their position on the floor, in a situation more favorable for the access of asphyxia.

7. *What quantity of charcoal would be required to be burnt in order to asphyxiate two adult individuals, in such an apartment as that of the prisoner?*

The reporter remarks that suicides, (judging from the published reports,) rarely consumed more than a bushel of charcoal to accomplish their purpose; and he argues, it was highly probable that a quantity less than this, would have sufficed to produce a fatal vitiation of air in the prisoner's apartment. The fatal effects of the vapour, however, depend almost as much on the rapidity with which the charcoal is burnt, as on the absolute quantity of fuel consumed. A question of this nature could, of course, only be answered approximatively. Thus much was certain: the prisoner, according to his own confession, had burnt a much larger quantity than was necessary to render the air of a confined room insupportable to life, and the combustion had been rapidly conducted. The last question proposed was:

8. *What quantity of ashes ought a given weight of charcoal to yield?*

This was to test the accuracy of the prisoner's statement, as to the quantity of charcoal which he alleged he had consumed; in other words, to discover whether he had thus really made an attempt on his own life after the death of the deceased. It was not possible to furnish any positive answer to this question, the quantity of ashes from burnt charcoal varying according to the nature of the wood, from which the charcoal has been made. A bushel of charcoal ought to yield a little more than one-sixteenth of its bulk of ashes.

The prisoner upon the evidence adduced, was found guilty of having caused the death of his wife, and was condemned to the gallies for life.

[The circumstantial evidence seems to leave but little doubt in this case, that the deceased had really perished from the effects of carbonic acid. The rapid supervention of putrefaction might be accounted for by the heat of the room: and the absence of livor on the body, by the discoloration which the skin had undergone from that process. It is scarcely possible to imagine that the prisoner remained in the room and made the repeated attempts to destroy himself by suffocation, in the manner urged in his defence. It is evident from a consideration of the whole of the circumstances, that his intention was to destroy his wife and to save himself. If he had used any violence to confine the deceased in the room, or if he had rendered her insensible prior to placing her there, it was not to be discovered by any external marks: or if such marks had existed, they had become subsequently effaced by the rapid putrefactive changes.] *Annales d'Hygiène. Janvier,* 1837.

---

### *On the Absorption of Sulphuric Acid, when introduced into the Stomach.* By A. Bouchardat and the late M. Cæsar Couriard.

It has been hitherto supposed that the mineral acids destroy life either by the local impression which they produce on the coats of the stomach, or by the inflammation and disorganization which they cause in that viscus. Since it is known that sulphuric acid speedily coagulates albumen and all albuminous liquids, it becomes difficult to understand how, when taken internally, this poison should become absorbed so as to react upon the blood; yet it is possible that the acid may enter and be prevented from combining with albumen, just as antagonist bodies may be intermixed and prevented from entering into combination, while exposed to the influence of an electric current. Or, allowing that an insoluble compound of acid and albumen is formed on its first absorption into the smaller vessels, it is possible to conceive that this compound may itself become absorbed, and react upon the blood in the larger trunks. But without resting the opinion upon mere suppositions, let us expose the facts which have induced us to admit that sulphuric acid may become absorbed into the circulating system.

1*st Observation.* A young woman, aged eighteen, swallowed, as it was conjectured, about an ounce of the sulphate of indigo. Immediately afterwards she felt an acute pain in the throat and in the stomach. She threw herself on the ground, and her cries soon brought her neighbours, who found her vomiting a bluish-coloured liquid, which effervesced on the pavement. A quantity of oil and milk was immediately exhibited: the milk was speedily thrown up coagulated and of a blue colour. When brought to the hospital three hours afterwards, she was in the following condition: Her face was pale, her features somewhat altered, her eyes were sunk, and her lips of a violet tinge. There was a yellowish coloured spot on the upper lip at each angle of the mouth. The tongue was blue, and the throat was painful, with a strong sense of constriction. The epigastrium was tender; but there was no pain in the abdomen. The respiration was difficult, there were great anxiety, coldness of the upper extremities, and a quick and small pulse. Her intellect was clear, and her answers to the questions put were sensible and proper. Four drachms of calcined magnesia in a pint of water were administered. A great portion of this mixture was rejected by vomiting, accompanied by bluish clots. In a few hours the pain in the throat became very severe, the upper extremities cold, and the pulse imperceptible. The urine which she passed had a *slight tinge of blue.* She continued to become worse, vomiting of chocolate-coloured matter supervened; and she died about *eleven* hours after having taken the poison.

The body was examined twenty-seven hours after death. The head presented no particular appearance. There was no sign of corrosion in the mouth. The mucous membrane of the pharynx and œsophagus was easily detached in dry white brittle layers. The heart was filled with three ounces of *coagulated blood;* and the aorta contained brown and semi-liquid *clots.* The lining membrane of this vessel was of a bright red colour. The stomach was distended, containing two ounces of a dark-coloured liquid. The mucous membrane was carbonized and of the colour of soot, intermixed with slight patches of redness throughout its whole extent, except for about an inch near the pylorus, where it was of a rose-red colour.

It was easily detached in layers, but there was no trace of ulceration. The lining membrane of the duodenum was inflamed and ulcerated, and in parts corroded and blackened. A dark-coloured mucus was found in the small intestines, and patches of a blue colour were scattered through the colon. The femoral arteries were filled with *semicoagulated dark-coloured blood.* The cavity of the left femoral artery was completely obstructed by the clot.

The whole progress of this case, taking the symptoms observed during life with the appearances found after death, justifies us in concluding that the deceased was not destroyed by the directly corrosive action of the poison on the digestive organs, but indirectly by the coagulation of blood in the circulating system. We endeavoured to corroborate the evidence furnished by this case by some experiments performed on dogs; but we found it difficult to say on these occasions, whether death was owing to the direct or indirect action of the poison. When very diluted sulphuric acid is given to dogs, there is no more poisonous action than when any ordinary acid liquid containing sulphuric acid is given to a human being. In these instances the action of the acid is superficial, it does not appear to become absorbed.

2*d Observation.* In October 1835, a young woman presented herself at the Hôtel Dieu, complaining of having suffered much from some medicine which she had taken the day preceding. On examination, a dark yellow spot was seen on her face near the commissure of the lips, her tongue and the mucous membrane of the mouth were highly inflamed, and the act of deglutition was painful. She was strictly questioned as to whether she had attempted to poison herself, but this she positively denied. It was evident that she was in a state of pregnancy, but this she also denied. The prescription of the medicine which she had taken directed a mixture of four drachms of sulphate of magnesia in three ounces of chamomile water; but when the bottle was examined, it was found to contain two drachms of a liquid consisting of equal parts of sulphuric acid and water. Some gum-water was administered to her which she continued to swallow with great difficulty; but it was almost instantly rejected. Her pulse was small and frequent: there was scarcely any burning sensation in her stomach, and indeed her only complaint was of pain in her throat. After a quiet night she was found much more composed, and could swallow more freely. Her pulse, however, was depressed, and her extremities were cold. During the second night she suffered from violent cramps in the lower extremities and of an entire loss of feeling in her right leg, which was found to be extremely cold at its lower part. On the following morning the symptoms abated; but there was no return of sensation in the right leg, her pulse became weaker and weaker; and on the third night after her admission she died without appearing to suffer.

On inspection, the mucous membrane of the œsophagus was found of a yellowish-black colour: it was covered with a similarly-coloured liquid, which was so adherent to it as almost to resemble a false membrane. The lining membrane was thickened, but was easily detached in layers. About five ounces of a dark yellowish liquid were found in the stomach. The mucous membrane of the viscus was in the same condition as that of the œsophagus; and its surface was covered with a dark yellowish mucus, which was remarkably firm and strongly adherent at the pyloric extremity. On removing a portion of this, the membrane beneath was found as it were carbonized. The duodenum and the small intestines presented a similar appearance. The heart contained about *three ounces of coagulated blood,* the aorta was almost filled with *gelatinous clots;* and the femoral artery of the right leg was completely obliterated by a cylinder of *dark coloured* and *firm coagula.* [The liquid found in the stomach and the *coagula* of blood contained in the femoral artery were submitted to an elaborate analysis, which we must here omit. The result will appear from the observations which follow.]

This evidence of the presence of sulphuric acid in the blood is not very satisfactory; for there were only *traces* of sulphate of barytes, and merely the odour of sulphurous acid was perceived: but if to these results we add the symptoms observed during life, the coldness of the extremities, the death of the lower extremity before general death, and the presence of a coagulum obstructing the femoral artery, we have reason to conclude that, in both of the observations reported, death was to be ascribed to the coagulation of the blood by the action of sulphuric acid.

The above facts show that sulphuric acid may destroy life in two ways, 1, by its direct action: 2, by absorption. In the first case the membranes of the alimentary canal are deeply carbonized and corroded, death is preceded by an acute gastro-enteritis and peritonitis, the stomach and intestines are distended with gas, and death ensues in the midst of the acutest suffering. If the sulphuric acid were combined with indigo the colouring matter does not appear in the urine: such are the characters of *local* poisoning. In the *second* case when sulphuric acid is *absorbed,* the local suffering is inconsiderable and the abdomen is not distended. There is no peritonitis, the pain in the epigastrium diminishes, and there is an appearance of recovery; but the pulse sinks, the extremities become cold, painful cramps supervene, and death takes place at a time when the local symptoms would lead us the least to expect that event. If this view of the operation of sulphuric acid be correct, the treatment of poisoning by this body ought to undergo some modification. Thus, instead of administering calcined magnesia, it would be proper to employ the bicarbonate of potash or soda, either of which is rapidly absorbed. By their entrance into the circulation, these alkaline salts may have the effect of destroying the coagula which might have been produced by the acid.

[REMARKS. We need hardly say that this paper is worthy of attention from the novelty of the views which are displayed in it. Sulphuric acid, like the other mineral acids, has been considered by toxicologists to operate independently of absorption, for the following reasons: 1. Dilution with water promotes the absorption and consequently accelerates the operation of many poisons, but dilution destroys entirely the effect of sulphuric acid in depriving it of its corrosive properties. 2. If absorbed, the acid would immediately obstruct the smaller vessels by coagulating the blood contained in them. The vital properties of a part are not sufficient to prevent this chemical action, since it is an effect every now and then witnessed in the gastric vessels as a result of the action of the acid through the coats of the stomach. 3. The acid has never been detected in the blood of persons poisoned by it. Strong as these reasons appear for the non-absorption of sulphuric acid, they cannot of course prevail against any new facts which may lead to a contrary presumption. Still the evidence against the present doctrine ought to be unequivocal; since, as the authors of the paper suggest, an alteration in our views respecting the operation of such poisons may materially modify the mode of treatment adopted. We must, however, maintain that the cases here detailed do not in our judgment present any such facts.

Although we do not pretend to deny the *possibility* of a mineral acid, like the sulphuric, entering into the blood by absorption, (indeed this may be necessary to its ordinary medicinal operation,) yet we contend the point still remains unsettled, and it will so remain, until analyses of a less equivocal nature establish its presence in the blood of persons who have taken it. Sulphuric acid forms a very intimate combination with albumen; and it would be by no means difficult to detect it in a coagulum where it really had existed in sufficient quantity to produce coagulation. In the mean time the hypothesis of the authors of this paper involves this parodox: the acid is capable of traversing the minute capillaries without coagulating their contents or blocking up their canals, while it recovers its coagulating power on the blood when it reaches the heart, aorta, and larger arteries. It will render the femoral artery impermeable by a consolidation of its contents, after having had its coagulating power suspended in the numerous vessels through which it must have passed before it could have reached that artery.]

*Annales d'Hygiène. Avril,* 1837.

